# Automatic Identification of Alpine Mass Movements by a Combination of Seismic and Infrasound Sensors

**DOI:** 10.3390/s18051658

**Published:** 2018-05-22

**Authors:** Andreas Schimmel, Johannes Hübl, Brian W. McArdell, Fabian Walter

**Affiliations:** 1 Institute of Mountain Risk Engineering, University of Natural Resources and Life Sciences (BOKU), 1190 Vienna, Austria; johannes.huebl@boku.ac.at; 2 Swiss Federal Institute for Forest, Snow and Landscape Research WSL, 8903 Birmensdorf, Switzerland; brian.mcardell@wsl.ch; 3 Laboratory of Hydraulics, Hydrology and Glaciology (VAW), ETH Zürich, 8093 Zürich, Switzerland; walter@vaw.baug.ethz.ch

**Keywords:** infrasound sensors, seismic sensors, debris flow, detection system, identification system

## Abstract

The automatic detection and identification of alpine mass movements such as debris flows, debris floods, or landslides have been of increasing importance for devising mitigation measures in densely populated and intensively used alpine regions. Since these mass movements emit characteristic seismic and acoustic waves in the low-frequency range (<30 Hz), several approaches have already been developed for detection and warning systems based on these signals. However, a combination of the two methods, for improving detection probability and reducing false alarms, is still applied rarely. This paper presents an update and extension of a previously published approach for a detection and identification system based on a combination of seismic and infrasound sensors. Furthermore, this work evaluates the possible early warning times at several test sites and aims to analyze the seismic and infrasound spectral signature produced by different sediment-related mass movements to identify the process type and estimate the magnitude of the event. Thus, this study presents an initial method for estimating the peak discharge and total volume of debris flows based on infrasound data. Tests on several catchments show that this system can detect and identify mass movements in real time directly at the sensor site with high accuracy and a low false alarm ratio.

## 1. Introduction

As described by [1], debris flows are highly concentrated mixtures of water and fine and coarse sediments, often containing additional woody debris, with front velocities of up to 20 m/s. The coarse sediment is usually concentrated in the upper layers and at the front of the flow, and the sediment concentration is often between 40% and 70% by volume with a specific bulk density of 1.7–2.4 g/cm${}^{3}$. Mudflows are similar to debris flows, but have a smaller concentration of boulders and more fine material [2]. Debris floods have higher water content and a finer sediment concentration in comparison to debris flows. Compared to debris flows and debris floods, bedload transport processes have no defined front and also have a greater water content.

Due to the fast socio-economic development of mountain areas, processes such as debris flows, debris floods, and bedload transport constitute an increasing hazard to lives and property. Monitoring debris flow torrents is essential for warning purposes and for gaining more knowledge about the processes. The fact that such mass movements emit seismic and acoustic waves in the low-frequency range (<30 Hz) enables detection of these events from a safe location unaffected by the process. Consequently, several approaches for detection and warning systems based on seismic or infrasound signals have already been developed.

The main source of seismic energy delivered by debris flows is the basal friction of the dense body of the flow in contact with the channel bed and boundaries (e.g., [3,4]). Seismic signals are less affected by wind and weather than are infrasound signals, but they have a strong dependency on the modulus (stiffness) of the ground and the distance between channel and sensor [5]. The monitoring of mass movements with geophones or seismometers is quite common [6,7,8,9] and a range of detection systems based on seismic signals has already been designed (e.g., [10,11,12]).

Infrasound waves are produced by the turbulent flow part of the mass movement [13,14]. These acoustic waves are detectable over large distances (several km) due to the frequency-dependency of atmospheric attenuation, which absorbs high-frequency (audible and ultra-) sound more than infrasound (<20 Hz) [15]). Infrasound signals produced by mass movements are generally in a relatively noise-free band in the low-frequency acoustic spectrum (≤20 Hz) where the main noise is induced by wind [16]. Several detection systems for sediment-related transport processes based on infrasound signals have been developed [17,18], although these methods are less common than those based on seismic signals.

Since both seismic and infrasonic mass movement monitoring have benefits and drawbacks, research on the combining of both technologies has been conducted in recent years. Both [19] and [16] show the correlation between seismic and infrasound signals from avalanches and debris flows and [20] analyzed the signal pattern of both for debris flows and how they can be used for a detection method.

For the identification of an event, different methods for signal classification have already been analyzed. An approach was developed by [21] for seismic detection of snow avalanches based on the fusion of data derived from the analysis of the signal in three domains: time, time frequency and polarizations. A database of observed avalanches was used by [22] to derive ten characteristic parameters that are determined from the time series and power spectrum. These parameters are used for automatic detection. The Hilbert–Huang transform (HHT) approach was applied by [17] to analyze the infrasound and geophone signals induced by debris flows.

This paper describes an extension of the already published approach for an early warning system based on the combination of seismic and infrasound sensors [23]. The recorded signals are automatically analyzed by a detection algorithm in order to identify events in real time as soon as possible, while at the same time reducing the number of false detections. We present updates made in the new version of the detection algorithm and we provide a deeper insight into the hardware setup and software design. Since the number of test sites has nearly doubled in recent years, we now can present the detection results from a large number of new recorded events and analyze the possible average early warning times of this system. Furthermore, an evaluation of the possibility to estimate the discharge based on infrasound or seismic signals has been carried out, and we present here a method to estimate the peak discharge and the total volume of an event based on infrasound signals.

## 2. Detection System

This section will describe the updates made on the detection system presented in [23] and will give a deeper insight into the hardware and software design. The basic setup of a combination of one seismic and one infrasound sensor together with a microcontroller has not been modified, but the detection algorithm has been updated and new sensors has been tested. As already mentioned in [23,24], this system is low cost and easy to install, so that it can be extended to a warning system for different applications: safeguarding of traffic routes by controlling traffic lights, protection of construction sites inside torrents (e.g., for cleaning up a basin after an event), or at sites where the necessary funding for expensive torrent and avalanche barriers is not available.

### 2.1. Hardware Setup

Three different infrasound sensors and two different geophones are used for the current setup. An example of an infrasound sensor used at three test sites is the Chaparral Model 24 (Chaparral Physics, Fairbanks, AK, USA), which has a sensitivity of 2 V/Pa and a frequency range from 0.1 to 200 Hz. As a second sensor, we use the infrasound microphone MK-224 with a frequency range from 3 Hz to 200 Hz and a sensitivity of 50 mV/Pa. Both sensors had already been used in [23] but proved to be rather expensive. Thus, a cheaper alternative was found with an Electret microphone of the type KECG2742WBL-25-L (Kingstate Electronics Corp., Taipei Hsien, Taiwan). Due to the use of this microphone, the overall system costs can be reduced by a factor of six compared to the system cost using a Chaparral sensor. The Electret microphone has a sensitivity of −38 dB ± 3 dB in the frequency range used, which results in approximately 13 mV/Pa and a typical frequency range of 20 Hz to 20 kHz. Because we use this microphone below 20 Hz and it has a large variation in sensitivity, it has to be calibrated, which is done by comparing the signal with the Chaparral Model 24. As seismic sensors, we use the Sensor NL SM-6 (ION, Leiden, The Netherlands) with a sensitivity of 28.8 V/m/s and a natural frequency of 4.5 Hz in addition to the Sercel SG-5 geophone (Sercel Inc., Houston, TX, USA) used formerly with a sensitivity of 80 V/m/s and a natural frequency of 5 Hz.

**Figure 1:**
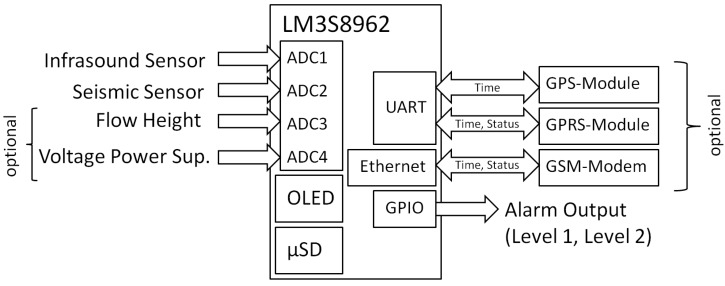
Overview system setup and components.

The sensor signals have to be adapted for the input of the microcontroller, which is done by a non-inverting OPV circuit (except for the Chapperal Model 24, where an inverting OPV circuit is used). This input circuit also has a band pass filtering with a lower cut-off frequency of around 150 mHz (for acoustic sensors) and an upper cut-off frequency of 150 Hz (acoustic and seismic sensors) included. These input signals are sampled by the microcontroller ADC (analog-to-digital converter) with a sample rate of 100 Hz, whereby a 32× hardware oversampling is used to avoid aliasing. A Stellaris Evaluation Board with the microcontroller LM3S8962 (Luminary Micro Inc., Austin, TX, USA) is used for the data processing and as data-logger. This microcontroller is based on the 32-bit ARM Cortex-M3 architecture with a processor frequency of 50 MHz, 64 KB RAM and a Flash storage of 256 KB. The evaluation board has four ADCs, two UARTs (Universal Asynchronous Receiver Transmitters), several GPIOs (general purpose input/output), which can be used as alarm outputs, and an OLED (organic light emitting diode) display and offers the possibility of an Ethernet connection. The data can be stored on a micro SD card where up to four months of data can be recorded continuously on a 16 GB card with the file structure as described in Section 2.2. Besides the input of the sensor signals, the free ADCs offer the possibility to log the flow height measured by a radar or ultrasonic gauge, which can be used for event verification. In addition, the power supply voltage can be measured to check for low power situations. If the test site is equipped with a standard internet connection, the communication with the system can be conducted via the Ethernet interface. If there is no router available, we use a GSM module of the type SparqEE CELLv1.0 [25], which is controlled by means of UART by AT commands. The time synchronization of the station is done by either a connection with a time server via Ethernet, or by a GPS module, which is also connected via the UART. An overview of the hardware components and the inputs and outputs of the system is given in Figure 1.

The LM3S8962 operates at a voltage of 5 V, provided by a DC–DC converter, which needs a power connection at a voltage range from 6.5 to 32 V. The system has a power consumption below 1.5 W, which makes this system very useful for stand-alone stations using a solar power supply, as is typically used. The minimum solar power supply designed for this system should be based on a 12 Wp solar panel and a 16 Ah battery. This specification might have to be adapted due to sun availability or communication setup.

### 2.2. Software Design

The software for the microcontroller was written in C and is based on the open source runtime system FreeRTOS [26]. FreeRTOS offers the possibility to create several tasks with different priorities and cycling times. Six different tasks are used for this system: the task with the highest priority is the measurement task, where the input signals are sampled at a sample rate of 100 Hz, and then converted and stored in an array. This array offers the input for the detection task, which is executed every second. This detection task is responsible for the signal processing (fast Fourier transform (FFT)) and runs the detection algorithm described in Section 2.3. For the storage of the data to the micro SD card, a log task is run every second. Three different files are created by this task: the raw data of the infrasound and seismic sensor are written into one text file and the output of the detection algorithm and ancillary data, such as flow height measured with an additional sensor and power supply voltage, are written in another file. Both files are created every hour. Another file stores a summary of all log files, and displays event detections or errors. A time task is created for the control of the system time. A control task regulates the alarm outputs, the point in time for output messages and time synchronization, and reacts to the inputs from the evaluation board keys. The output messages and the time synchronization are done as part of a communication task where the Ethernet connection is used via LWIP (light-weight implementation of the TCP/IP protocol) or the GSM module is controlled via UART. The system is designed to send a status message to a server every hour, whereby the date of the event detections or error messages are included. This server creates e-mail alerts in the case of an event. A web server is installed on this server as well, where the status and events at all stations can be checked [27]. Table 1 shows a list of the tasks performed, cycling time and their priorities in the FreeRTOS runtime.

### 2.3. Detection Algorithm

Based on the results at the test sites, the detection algorithm presented earlier in [23] has been modified to increase the detection probability and reduce the frequency of false alarms. This section will give a short summary of the (new) detection algorithm and its different criteria.

As the former version, the input signals that are sampled with 100 Hz are processed by fast Fourier transform (Bluestein FFT algorithm [28]) with 100 samples every second, and different frequency bands of the infrasound and seismic signals have to fulfill several detection criteria for a specific time span (detection time). Three different criteria are applied to the infrasound signal: for the first criterion, the average amplitude of a debris flow (${\bar{A}}_{\mathrm{DFlow}}$) or debris flood (${\bar{A}}_{\mathrm{DFlood}}$) frequency band has to exceed a threshold for the detection time. We use two different frequency bands for debris flow (3–15 Hz) and debris flood signals (15–45 Hz) because the peak frequency of the infrasound signals depends on the viscosity of the event (e.g., [16]). Thus, these two frequency bands represent the typical signals of debris flows and debris floods and can be used for process-type identification. Two limits are used to distinguish between different event sizes. Detections fulfilling the Level 1 threshold $A_{\mathrm{LimitL}1}$ are mostly higher discharge with sediment transport or small debris floods; event detections at Level 2 (threshold $A_{\mathrm{LimitL}2}$) are typically “fully developed” debris flows and debris floods:

(1) $$\mathrm{Level}\;1:\;\;{\bar{A}}_{\mathrm{DFlow}}\geq A_{\mathrm{LimitL}1}\;\;\mathrm{or}\;\;{\bar{A}}_{\mathrm{DFlood}}\geq A_{\mathrm{LimitL}1},$$

(2) $$\mathrm{Level}\;2:\;\;{\bar{A}}_{\mathrm{DFlow}}\geq A_{\mathrm{LimitL}2}\;\;\mathrm{or}\;\;{\bar{A}}_{\mathrm{DFlood}}\geq A_{\mathrm{LimitL}2}.$$

As a second criterion, the average infrasound amplitudes of the debris flow or debris flood frequency band have to be at least above a third (for debris flows) or a fourth (for debris floods) of the amplitudes of the frequency band below (${\bar{A}}_{\mathrm{low}}$):

(3) $${\bar{A}}_{\mathrm{DFlow}}>\frac{{\bar{A}}_{\mathrm{low}}}{3}\;\;\mathrm{or}\;\;{\bar{A}}_{\mathrm{DFlood}}>\frac{{\bar{A}}_{\mathrm{low}}}{4}.$$

These criteria serve mainly to prevent false alarms due to wind that dominates this low-frequency band. The wind-produced amplitudes in this low-frequency band (≤2 Hz) are usually at least three times higher than the amplitudes in the debris flow frequency band and at least four times higher than the amplitudes in the debris flood frequency band. In the former version [23,29], a higher frequency band was also used, whose amplitudes had to be lower than the debris flow/debris floods bands’ amplitudes. Because debris floods with higher peak frequencies have not been detected by the algorithm including this criterion, it has been omitted and a greater detection time was chosen in place of this criterion.

The variance in amplitudes in the debris flow or debris flood frequency band is used by the third criterion of the detection algorithm. Since this variance in the amplitudes of the broad-banded debris flow or debris flood signals is low, compared to narrow-banded signals from artificial sources (such as aircrafts, cars, machines, etc.), this criterion efficiently reduces the frequency of artificially caused false alarms. Therefore, the variance in the amplitude $A_{\mathrm{VarIS}}$ has to be under a certain limit ($A_{\mathrm{VarLimit}}$):

(4) $$A_{\mathrm{VarIS}}\leq A_{\mathrm{VarLimit}}.$$

Only the amplitude and the variance criteria are used for the seismic signals. Since the dependency of peak frequencies on the viscosity is not as significant as for infrasound signals, only one frequency band is used for debris flows and also for debris floods (${\bar{A}}_{\mathrm{DFlow}/\mathrm{DFlood}}$). For the classification of the event size, two different limits ($A_{\mathrm{LimitL}1}$, $A_{\mathrm{LimitL}2}$) are also used:

(5) $$\mathrm{Level}\;1:\;\;{\bar{A}}_{\mathrm{DFlow}/\mathrm{DFlood}}\geq A_{\mathrm{LimitL}1},$$

(6) $$\mathrm{Level}\;2:\;\;{\bar{A}}_{\mathrm{DFlow}/\mathrm{DFlood}}\geq A_{\mathrm{LimitL}2},$$

(7) $$A_{\mathrm{VarGEO}}\geq A_{\mathrm{VarLimit}}.$$

Because bedload transport processes as well as debris flows and debris floods can be detected, a further criterion is needed to enable identification of event type. Debris flows and debris floods typically occur in several surges and have a well-defined front compared to bedload transport (e.g., [30]), so these event types can be identified by a rapid rise in the seismic or infrasound signal. For debris flow/debris flood detection, the seismic amplitude has to rise at least beyond the threshold used for the amplitude criterion during the detection time, whereby we also distinguish between Level 1 ($A_{\mathrm{LimitL}1}$) and Level 2 ($A_{\mathrm{LimitL}2}$) detections. Because the signal sequence of the seismic amplitudes is smoother than that of the infrasound signals, we use seismic amplitudes for this identification criterion.

**Figure 2:**
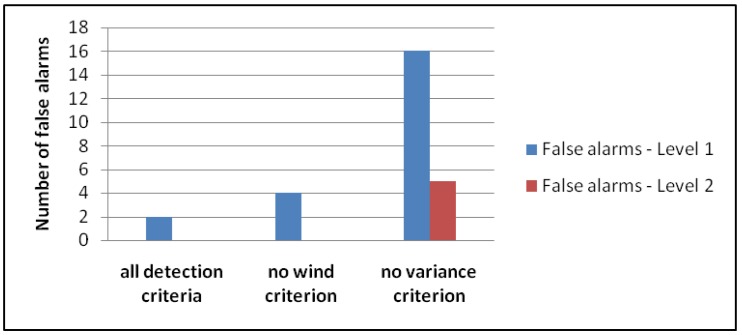
Effect of the different detection criteria on the false alarms at the Gadria test site in 2016.

These criteria have to be fulfilled for the infrasound as well as for the seismic signals for the detection time threshold of 20 s to trigger an event detection. Several actions of the warning system can be started based on the two alarm levels (e.g., SMS alert, control of a traffic light, etc.), depending on the application. With the combination of the seismic and infrasound signals, we achieve a high detection ratio and a strong reduction in the frequency of false alarms. For example, at the Gadria test site in South Tyrol in 2016, the system would register more than one hundred false alarms if either the infrasound or the seismic data were used alone. By combining both technologies, and applying all criteria, the false alarms can be reduced to only two. If we omit the wind criterion (Equation (3)), the false alarms will double, and if we omit the variance criterion (Equation (4)), the false alarms will increase to 21 (Figure 2).

The parameters of the detection algorithm (Table 2) have been identified in an exhaustive evaluation and optimization process and show good results at all test sites. Compared to the first version presented in [23], we have adjusted the frequency bands of the infrasound signals in the low-frequency range to ensure better detection of extremely viscous debris flows that have their peak amplitudes in a low frequency range (<5 Hz, [31]). Furthermore, the upper limit for the debris flood frequency band has been increased to enable better detection of certain debris floods with higher peak frequencies. For further applications of the system, the parameters can be adapted to special requirements of the site, application and the background noise.

## 3. Test Sites

**Figure 3:**
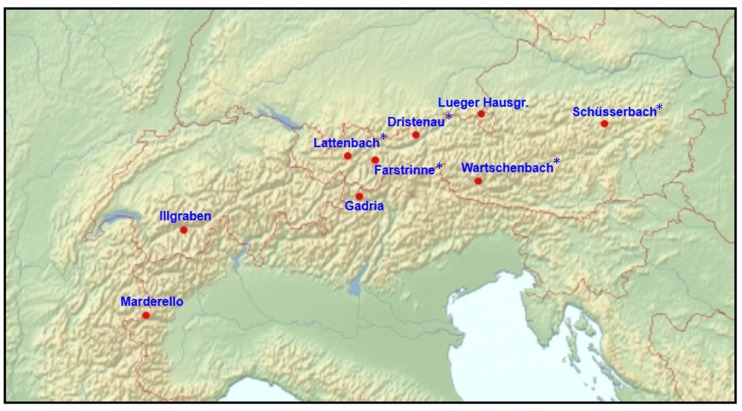
Overview of the test sites from 2013 to 2016 (* test sites already presented in [23]).

Over the past several years, several torrent catchments have been instrumented with the detection system described here (Figure 3). The Tyrolese torrents Lattenbach, Dristenau and Farstrinne [23] and the Schüsserbach in Styria were equipped with the system during the period from 2013 to 2016. The system was operational in 2013, 2015 and 2016, at the eastern Tyrolese test site Wartschenbach. The system has been running at Illgraben, Gadria and Marderello catchments since 2015, and the Bavarian Lueger Hausgraben torrent was equipped with the system in summer 2016.

The following section describes two examples of event detection at the Marderello and Illgraben test site.

## 4. Example Events

### 4.1. Marderello

**Figure 4:**
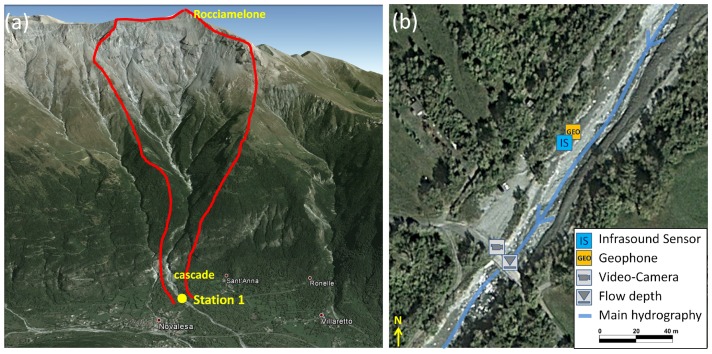
(**a**) overview of the test site Marderello (red line: catchment area); (**b**) closer view of the monitoring station and sensor setup (Source: Google Maps).

The Marderello catchment is located in the northwestern Italian Alps in the eastern part of the Cenischia valley. It has a catchment area of 6.61 km${}^{2}$, and the upper basin altitude is at 3538 m a.s.l. at the Rocciamelone Mt., extending down to the village of Novalesa at an altitude of 900 m a.s.l., with an average slope gradient of 60%. Due to its geomorphic conditions [32], frequent mudflows and debris flow events occur in this catchment. Initial monitoring activities began in 1994, and, in 2013, the monitoring system was improved. It is now equipped with one ultrasonic gauge for flow depth measuring, two video cameras and four geophones [33]. In the year 2015, the detection system based on an infrasound and a seismic sensor was installed in the lower part of the catchment close to the indicated monitoring Station 1 (Figure 4). A Chaparral Model 24 infrasound sensor and a Sercel SG-5 geophone were installed.

**Figure 5:**
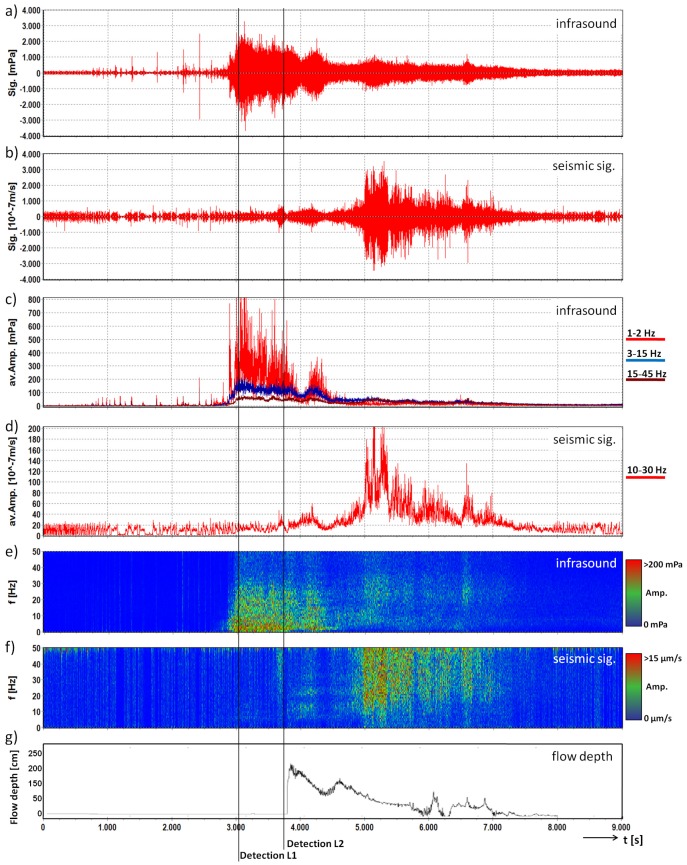
Infrasound and seismic data of the mudflow monitored at the Marderello test site on 9 August 2015. Signals are represented with a common base of time. (**a**) infrasound time series; (**b**) seismogram; (**c**) average amplitude of the three frequency bands of the infrasound signal; (**d**) average amplitude of the frequency band of the seismic signal; (**e**) running spectrum of the infrasound signal; (**f**) running spectrum of the seismic signal; (**g**) flow depth (∼70 m downstream); lines: time of first detection for Levels 1 and 2.

A mudflow at Marderello was recorded on 9 August 2015 beginning at 2:00 p.m. The total discharge of this mudflow was approximately 50,000 m${}^{3}$ with an event duration of around 4500 s. The infrasound and seismic signals of this event are shown in Figure 5. A significant feature of this event is the high amplitudes of the infrasound signal in the low frequency range (<5 Hz) between 2800 and 4400 s, while the seismic amplitudes are rather low in this time window. We assume that this phenomenon was due to the joint effect of (i) the passing of the mudflow at the waterfall in the upper area, between 3000 and 3500 s and (ii) the very low velocity during the first half of the flow, between 3500 and 4500 s. Waterfall-produced infrasound has been identified with peak frequencies below 5 Hz [34], and a possible misleading effect of the Marderello waterfall on the seismic signal was already observed for previous events [11].

The largest amplitudes of the infrasound signal occur at 3 Hz with 749 mPa and the maximum amplitude of the seismic signals was recorded at 23 Hz with 76 μm/s. The detection algorithm detects this event at 3048 s for Level 1 and 3688 s for Level 2. We use the flow height recorded by the ultrasonic gauge around 70 m downstream of the detection system and adapt this point in time according to the distance between the monitoring stations and the estimated velocity of the mudflow, to estimate the event arrival at the detection system at 3690 s. This results in an early detection time of 642 s at Level 1 and 2 s at Level 2. This long early detection time for Level 1 can be achieved due to the low flow velocity of this mudflow.

### 4.2. Illgraben

**Figure 6:**
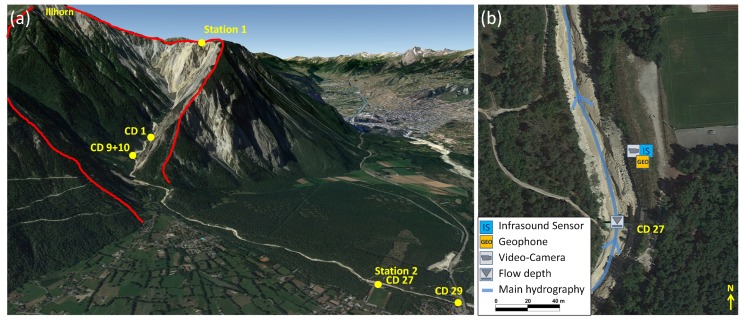
(**a**) overview of the test site Illgraben (red line: catchment area); (**b**) closer view of the monitoring station 2 and sensor setup (Source: Google Maps).

The Illgraben in Canton Vallis is the most active debris flow catchment in Switzerland. It has a catchment area of 9.5 km${}^{2}$ and extends from the summit of the Illhorn at 2716 m a.s.l. down to the Rhône valley at 610 m a.s.l. (Figure 6). The steep slopes in the upper catchment area and the highly fractured bedrock provoke frequent landslides and rockfalls, which results in abundant sediment deposits in the channel [35,36]. Due to effectively focused water runoff in the upper catchment area, these deposits lead to frequent debris flows and debris floods with an annual sediment discharge of several hundred thousand metric tons [37]. On average, three to five debris flows and debris floods are observed every year [38,39]. Since 2015, two of our detection systems have been installed at the Illgraben catchment. One of them is placed in the upper catchment area directly at the canyon rim (Station 1 in Figure 6a). This station is equipped with a Chaparral infrasound sensor and a SG-5 geophone. The other station (Station 2) is located down in the valley close to the channel mouth at check dam 27 near the football field. This station comprises an Electret microphone and a SM-6 geophone.

**Figure 7:**
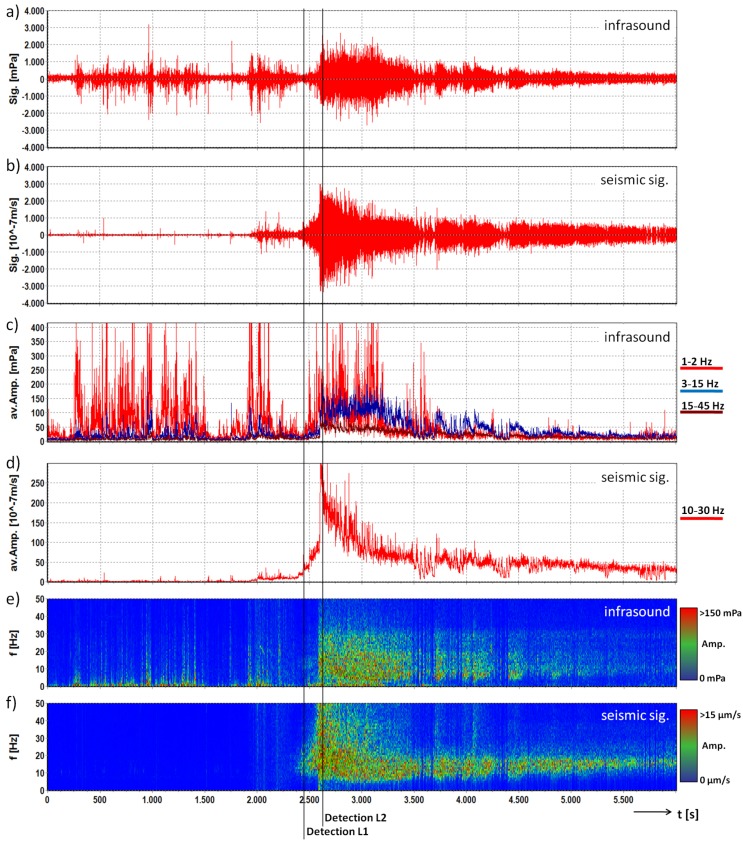
Infrasound and seismic data of the debris flow monitored at the Illgraben test site on 22 July 2015 at Station 2. Signals are represented with a common base of time. (**a**) infrasound time series; (**b**) seismogram; (**c**) average amplitude of the three frequency bands of the infrasound signal; (**d**) average amplitude of the frequency band of the seismic signal; (**e**) running spectrum of the infrasound signal; (**f**) running spectrum of the seismic signal; lines: time of first detection for Levels 1 and 2.

The debris flow illustrated here was recorded on 22 July 2015. Based on the installed measurement system [37], the event was classified as a small-sized debris flow with a total volume of 8700 m${}^{3}$, a peak discharge of 17 m${}^{3}$/s and a front velocity of around 2 m/s. Figure 7 shows the seismic and infrasound signals recorded at the Station 2 in the valley. The maximum infrasound amplitude of this event with 567 mPa occurred at 5 Hz, the maximum seismic amplitude of 119 μm/s was registered at 15 Hz and the event duration was approximately 3500 s. The high amplitude spikes in the first part of the infrasound signal (<2500 s) may be caused by cultural noise or a thunderstorm. The debris flow was detected by this station at 2462 s for Level 1 and 2617 s for Level 2. This results in an early detection time of 138 s (Level 1) before the event passes the sensor site (at 2600 s) and for Level 2 the detection occurred 17 s after the passing.

The existing early warning system at Illgraben (located at Check Dam 1 (CD 1), Check Dam 9 and 10 (CD 9 + 10), Check Dam 27 (CD 27) as well as at Check Dam 29 (CD 29) in Figure 6a; Ref. [39] provides a longer early warning time (up to several thousand seconds); however, that installation uses geophones installed at relatively exposed locations on the check dams in the channel as well as stage sensors (radar) suspended above the channel, and it relies on the mobile telephone network to transfer the alarms to warning lamps installed at several locations downstream. The system we describe here can be positioned at a safe distance from the channel. It may be possible to increase the early warning time by installing additional sensors closer to the source area, e.g., at the location of the detection sensors used by the Illgraben system, or by installing detection systems at each vulnerable channel crossing location. However, a more detailed comparison with the existing Illgraben early warning system is beyond the scope of this manuscript.

**Figure 8:**
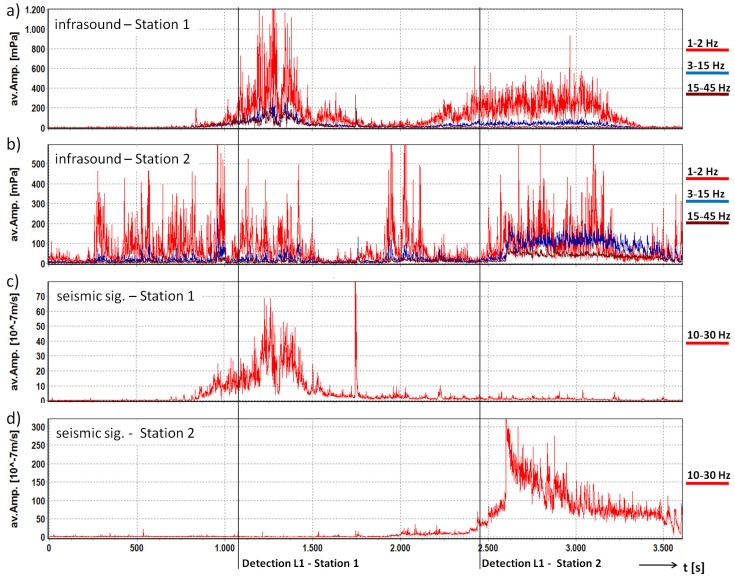
Comparison of the Infrasound and seismic data from the debris flow on 22 July 2015 at Stations 1 and 2 in the Illgraben catchment. Signals are represented with a common base of time. (**a**) average amplitude of the three frequency bands of the infrasound signal at Station 1; (**b**) average amplitude of the three frequency bands of the infrasound signal at Station 2; (**c**) average amplitude of the frequency band of the seismic signal at Station 1; (**d**) average amplitude of the frequency band of the seismic signal at Station 2; lines: time of first detection for Level 1 at Stations 1 and 2.

This debris flow was also detected at the upper Station 1. However, at this station, it has a different seismic/infrasound signature (Figure 8). The first part of the signal (1000 s to 1800 s) recorded at the Station 1 may have been caused by the initial process, which may have been a landslide in the upper catchment. This was not registered by the infrasound sensor at Station 2 in the valley because the infrasound was shielded by the mountain ridge. The second part (2200 s to 3400 s) of the infrasound signal may have been produced by the subsequent debris flow. This signal is also recorded later at Station 2. We suggest that the reason for the low-frequency infrasound signal recorded at Station 1 of the second part of the debris flow might be the frequency-dependency of atmospheric attenuation absorbing high-frequency sound more than low-frequency sound [15]. In contrast to the results of [40], which showed that the seismic signals of mass movements can be detected over the entire length of the channel, the seismic signals of the first time section could be only detected at Station 1 and the seismic signal of the debris flow was registered only at Station 2. We suggest that the geophones used are not sensitive enough to register the signals with such high magnitudes from sources at greater distances.

This example also shows that an installation from this system in the upper catchment area can identify initial processes and thereby significantly increase the early warning times compared to the setup down close to the channel. On the other hand, since the setup at Station 1 did not register all events, a combination of one station in the starting area and one in a lower part of the channel might offer the best solution.

## 5. Results and Discussion

### 5.1. Evaluation of the Detection Algorithm

For the evaluation of the detection algorithm, the numbers are analyzed from the registered events, false alarms and missed events for all test sites from 2013 to 2016 (Table 3). The events are divided into two different types: higher discharge with sediment transport and debris floods, and debris flows. To categorize the different events, several methods such as video or flow-depth measuring are used, but the available data did not allow a reliable distinction to be made between debris flow and debris flood at all test sites, so these are found in the same group. For the detections, we distinguish between Level 1 detections, which are found primarily in the category of sediment transport processes or small debris floods, and Level 2 detections, which are usually “fully developed” debris flows and debris floods. For some detections, it was not possible to classify the signals due to missing data for evaluation or other technical problems, so they are listed separately as “non-classifiable events”.

**Figure 9:**
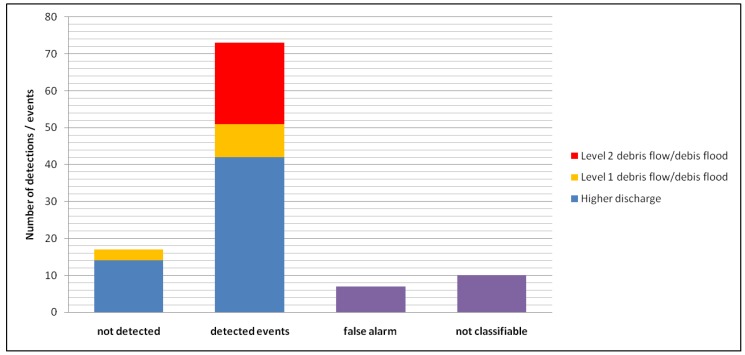
Summary of event detections and undetected events for all test sites from 2013 to 2016.

Most of the events from 2013 to 2016 were higher discharge processes (42), whereby 14 events could not be detected. Since most of the undetected events in this class were smaller ones, a detection of these events is not necessarily required, in contrast to the debris flow and debris flood events. The detection of these processes is obligatory and all 22 Level 2 events were detected and also nearly all Level 1 events were able to be detected (9 out of 12). During the entire operation time of 77,300 h, only seven false alarms were registered and 10 detections could not be classified clearly (Figure 9).

**Figure 10:**
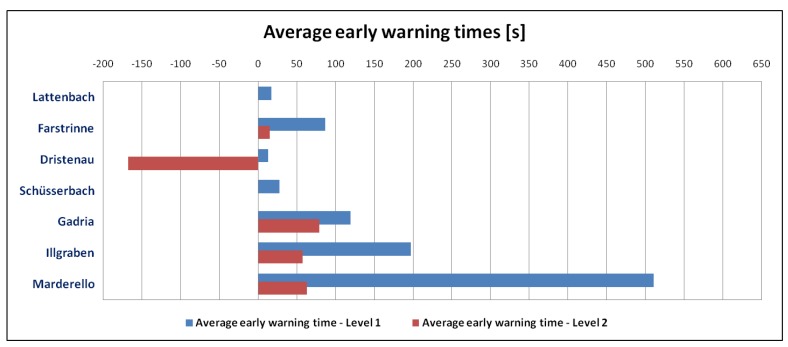
Overview average early warning times for all events listed in Table 4.

The time between the detection and the passing of the main surge at the sensor site (generally indicated by the maximum seismic and infrasonic amplitudes), which from here on is called “early warning time” is another factor that can be used to evaluate the system. Therefore, we compared the average early warning times of Level 2 events of the debris flow or debris flood type for all test sites (Table 4; Figure 10).

This evaluation shows the wide variance between the different test sites, whereby the average early warning times at Marderello are the longest at nearly ten minutes (at Level 1) compared to the average early warning times at Dristenau, where events are detected only a few seconds before passing. The Level 2 detections are closer to the passing of the main surge for most recorded events and, at the Dristenau test site, this time is even negative. This late Level 2 detection time is caused mainly by the smaller event size at this test site, where Level 2 detections are often results of later surges. Another reason is that, at the Dristenau test site, the position of the monitoring station is close to the valley end where events are generated, as described in [23].

### 5.2. Magnitude Estimation

**Figure 11:**
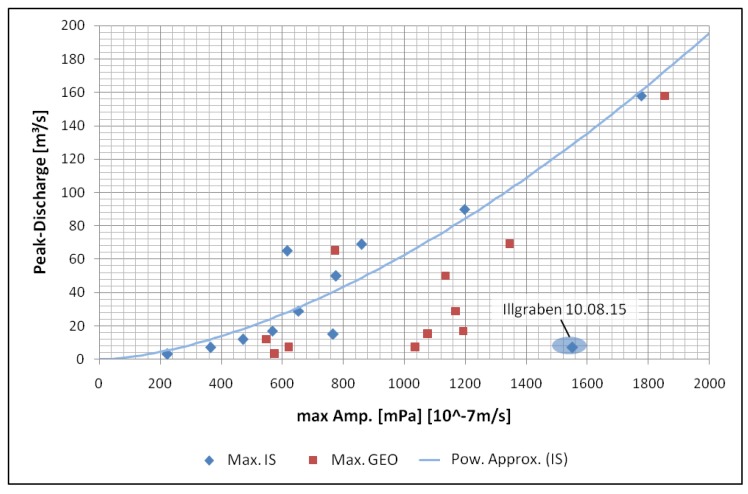
Peak discharge over maximum seismic (Max GEO) and infrasound amplitudes (Max IS) and the approximation based on infrasound data (Pow. Approx.).

We also estimated the event size based on the infrasound and seismic data. The infrasound and seismic energy correlates passably with the discharge of an event (e.g., [41]), so we compared the maximum infrasound and/or seismic amplitudes with the peak discharge of an event (Figure 11). The values for peak discharge and total volume used for this analysis are from Level 2 events at the Lattenbach, Gadria and Illgraben test sites (Table 5) and are calculated based on flow height measurements and velocity estimations. Since all monitoring stations used for this study are rather close to the channel (between 10 and 20 m) and the distances are nearly the same at every test site, we neglected attenuation of the signals in the air or in the ground, geometric spreading and the influence of topography or geology.

This analysis shows that, for peak discharge, the use of infrasound amplitudes with a power curve fitting offers a good approach to finding an initial relationship between the recorded signals and this event parameter. This curve fitting provides a $R^{2}$ of 0.955 for peak discharge. The approximation for peak discharge $Q_{\mathrm{peak}}$ (in m${}^{3}$/s) can be calculated based on the maximum infrasound amplitudes $A_{\mathrm{IS}(\max)}$ (in mPa) according to Equation (8). The marked outlier in the upper range of the maximum amplitudes is produced by the event on 10 August 2015 at the Illgraben test site and has not been included in the curve fitting process:

(8) $$Q_{\mathrm{peak}}=0.000732\;{A_{\mathrm{IS}(\max)}}^{1.644}.$$

**Figure 12:**
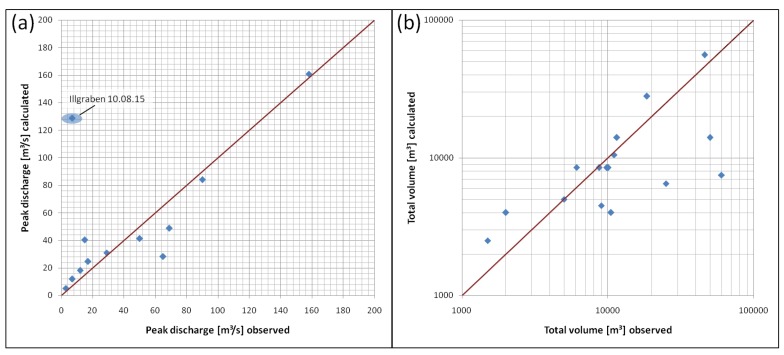
(**a**) comparison of the calculated peak discharge to the observed values; (**b**) comparison of the calculated total volume to the observed volume.

For an estimation of the total volume, we integrate the discharge calculated with the relationship for peak discharge (Equation (8)) over the entire detection time of an event. Figure 12 compares the calculated values (vertical axis) for peak discharge and total volume to the observed values (horizontal axis). The line represents the one-to-one relationship.

Both diagrams suggest that it may be possible to obtain first-order estimates of the peak discharge and the total volume for debris flows and debris floods at different sites based on the infrasound amplitudes. Calculation of the peak discharge based on infrasound data offers a good approximation ($R^{2}$ = 0.88), but, for the calculation of the total volume, this method shows a wide variance ($R^{2}$ = 0.27). Because the total volume is estimated by the sum over the event duration, this duration has to be defined based on the seismic and infrasound data, which is done by applying the detection criteria. Thus, the amplitude thresholds for the detection criteria also have an influence on the event duration and on the total volume estimation.

**Figure 13:**
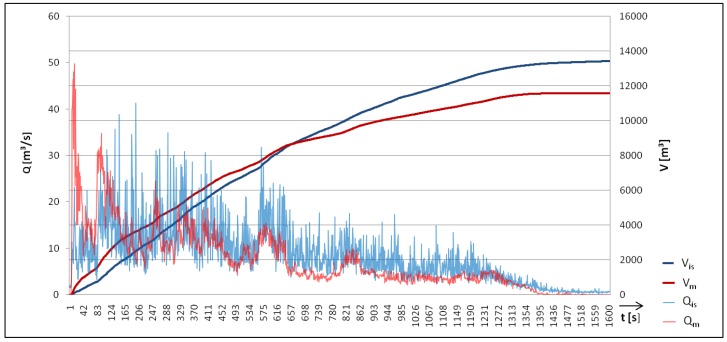
Calculated discharge (Q${}_{\mathrm{is}}$) and calculated volume (V${}_{\mathrm{is}}$) based on infrasound data compared to measured discharge (Q${}_{m}$) and measured volume (V${}_{m}$) of the debris flow at Lattenbach on 9 August 2015.

To evaluate this method for the magnitude estimation, we analyzed an event that occurred on 9 August 2015 at the Lattenbach test site. This debris flow with a total volume of 11,600 m${}^{3}$ and a peak discharge of 50 m${}^{3}$/s had a maximum infrasound amplitude of 776 mPa at 16 Hz and a maximum seismic amplitude of 113 μm/s at 29 Hz. If the method for the discharge estimation is applied to the infrasound signal, the peak discharge is calculated as 41 m${}^{3}$/s and the total volume as 13,430 m${}^{3}$. At the Lattenbach monitoring site, a 2D Laser scanner can be used in combination with a debris flow Puls–Doppler Radar (IBTP-Koschuch, [42]) for surface velocity to calculate, with a good degree of accuracy, the discharge of debris flows with a time resolution of one second during the whole event duration [43]. Figure 13 compares the discharge and total volume estimate based on the infrasound signal to the measured discharge. The total volume can be determined by summing up the discharge during the event time. This method results in an underestimate of the discharge based on the infrasound signal for the first time section of the debris flow and an overestimation in the second part of the debris flow duration.

This analysis showed that it is possible to achieve a rough estimate of peak discharge and total volume based on infrasound signals; nevertheless, further research and additional data on different events are necessary to develop a robust method for magnitude identification.

## 6. Conclusions

This paper demonstrates the potential for combining seismic and infrasound measurements to promote the development of an automatic rapid detection and identification system for debris-flow-related disasters. The proposed detection system based on one infrasound sensor, one co-located geophone and a microcontroller is inexpensive, portable and easy to install and can be extended to an early warning system for different kinds of alpine mass movements. As such, the combination of infrasound and seismic sensors increases detection probability and reduces the frequency of false alarms. It was possible to detect automatically all larger debris flows and debris floods in the period from 2013 to 2016 at nine different test sites, while only seven false alarms were registered in this time period. However, sensor equipment and installation location have to be chosen carefully (e.g., no shielding of infrasound, consolidated soil for the geophone, right position along the channel) and parameters of the detection algorithm may have to be adapted to the particular application and the background noise of the site. Initial analyses of different event types and different magnitudes have shown a dependency of the peak frequency range on the viscosity and a relation of the maximum infrasound and seismic amplitudes to the event magnitude. For this reason, a first estimate of the maximum discharge and the total volume based on the seismic or infrasound data is possible, whereby the infrasound amplitude seems to be a better approach for such a magnitude estimate. However, further research based on large databases of different well categorized events at different test sites is necessary for reliable event identification. An estimate of the process velocity could increase the accuracy of the identification of the magnitude and type since the infrasound and seismic signal signature also depends on the process velocity. Thus, as a further step, a method for velocity estimation would be included, which can be done by extending the system with an additional seismic of the infrasound sensor.

## Figures and Tables

**Table 1:** FreeRTOS tasks.

Task Name	Description	Priority	Time Interval
Measurement Task	Receives signals from ADC	6 (highest)	10 ms
Detection Task	Calculates FFT, execute detection algorithm	5	1 s
Time Task	Controls system time	4	1 s
Log Task	Data logging to SD card	3	1 s
Control Task	Controls outputs and points in time for com.	2	1 s
COM Task	Communication via Ethernet or UART	1 (lowest)	1 s (on demand)

**Table 2:** Current settings for the detection algorithm.

		Infrasound Signal	Seismic Signal
Frequency band 1	$FB1_{\mathrm{low}}$–$FB1_{\mathrm{high}}$	1 to 2 Hz	-
Frequency band 2—debris flow	$FB2_{\mathrm{low}}$–$FB2_{\mathrm{high}}$	3 to 15 Hz	10 to 30 Hz
Frequency band 3—debris flood	$FB3_{\mathrm{low}}$–$FB3_{\mathrm{high}}$	15 to 45 Hz	10 to 30 Hz
Limit for Amplitudes—Level 1	$A_{\mathrm{LimitL}1}$	12 mPa	1 μm/s
Limit for Amplitudes—Level 2	$A_{\mathrm{LimitL}1}$	30 mPa	2 μm/s
Limit for Variance	$A_{\mathrm{VarLimit}}$	0.8	
Time span for detection	$T_{\det}$	20 s	

**Table 3:** Events, detections and false alarms from 2013 to 2016.

				Debris Flow/Debris Flood							
		Higher Discharge		Level 1		Level 2		False Alarms		Not	Operating
Test Site	Year	Detected	Not Detected	Detected	Not Detected	Detected	Not Detected	Level 1	Level 2	Classifiable	Hours
Lattenbach	2013	0	1	0	0	0	0	0	0	1	4915
	2014	0	0	0	0	0	0	1	0	0	6184
	2015	0	1	0	0	3	0	0	0	0	3828
	2016	1	2	0	0	1	0	0	0	0	3620
Dristenau	2013	6	2	0	0	0	0	0	0	0	2202
	2014	0	0	1	1	0	0	0	0	0	2832
	2015	5	1	2	1	2	0	1	0	0	2907
	2016	14	2	1	0	0	0	0	0	0	3191
Farstrinne	2013	0	0	0	0	0	0	0	0	0	3488
	2014	1	0	0	0	1	0	0	0	0	4026
	2015	0	0	0	0	1	0	0	0	1	3648
	2016	0	0	1	0	0	0	0	0	0	3470
Schüsserbach	2013	1	2	0	0	0	0	0	0	0	1026
	2014	0	0	0	0	0	0	0	0	0	1365
	2015	0	0	2	0	0	0	0	0	0	2227
	2016	0	1	0	0	2	0	0	0	0	3470
Wartschenbach	2013	1	1	0	0	0	0	0	0	0	1771
	2015	2	0	0	0	0	0	1	0	4	2662
	2016	2	0	0	0	0	0	0	0	1	3473
Illgraben	2015	2	0	0	0	4	0	1	0	0	2161
	2016	1	0	0	0	5	0	1	0	0	2705
Gadira	2015	0	0	1	0	0	0	0	0	0	2351
	2016	1	0	1	0	1	0	2	0	0	2804
Marderello	2015	2	1	0	1	1	0	0	0	1	3023
	2016	3	0	0	0	1	0	0	0	2	2201
Lueger Hausgraben	2016	0	0	0	0	0	0	0	0	0	1750
	SUM:	42	14	9	3	22	0	7	0	10	77,300

**Table 4:** Average early warning times for Level 2 debris flow/debris flood at different test sites.

	Average Early Warning Time Level 1 (s)	Average Early Warning Time Level 2 (s)	Number of Events
Lattenbach	17	1	5
Farstrinne	87	15	2
Dristenau	13	−168	4
Schüsserbach	28	0	2
Gadria	120	79	3
Illgraben	197	57	8
Marderello	511	63	2

**Table 5:** Peak discharge and total volume.

Test Site	Event Date	Peak Discharge (m${}^{3}$/s)	Total Volume (m${}^{3}$)
Lattenbach	9 August 2015	50	11,500
	10 August 2015	69	18,500
	16 August 2015	12	5000
	10 September 2016	158	46,000
Gadria	15 July 2014	na	10,500
	8 June 2015	na	9850
	12 July 2016	na	1500
Illgraben	22 July 2015	17	8700
	10 August 2015	7	6100
	14 August 2015	7	25,000
	15 August 2015	3	2000
	12 July 2016	15	10,000
	12 July 2016	65	60,000
	22 July 2016	50–90	>10,000
	9 August 2016	29	<10,000
